# Multiplexed bulk and single-cell RNA-seq hybrid enables cost-efficient disease modeling with chimeric organoids

**DOI:** 10.1038/s41467-024-48282-5

**Published:** 2024-05-10

**Authors:** Chen Cheng, Gang Wang, Yuqing Zhu, Hangdi Wu, Li Zhang, Zhihong Liu, Yuanhua Huang, Jin Zhang

**Affiliations:** 1 Center for Translational Stem Cell Biology, Hong Kong Science and Technology Park, Hong Kong SAR, China; 2 Liangzhu Laboratory, Zhejiang University School of Medicine, Hangzhou, China; 3 School of Biomedical Sciences, Li Ka Shing Faculty of Medicine, The University of Hong Kong, Pokfulam, Hong Kong SAR, China; 4 Center for Stem Cell and Regenerative Medicine, Department of Basic Medical Sciences, and Bone Marrow for Transplantation Center of the First Affiliated Hospital, Zhejiang University, Hangzhou, China; 5 National Clinical Research Center of Kidney Diseases, Jinling Hospital, Nanjing University School of Medicine, Nanjing, China; 6 Department of Basic Medical Sciences, Zhejiang University School of Medicine, Hangzhou, China; 7 Center for Stem Cell and Translational Medicine, School of Life Sciences, Anhui University, Hefei, Anhui China; 8 Department of Statistics and Actuarial Science, The University of Hong Kong, Pokfulam, Hong Kong SAR, China; 9 Center of Gene/Cell Engineering and Genome Medicine of Zhejiang Province, Hangzhou, China

**Keywords:** Computational models, Kidney diseases, RNA sequencing, Lab-on-a-chip, Induced pluripotent stem cells

## Abstract

Disease modeling with isogenic Induced Pluripotent Stem Cell (iPSC)-differentiated organoids serves as a powerful technique for studying disease mechanisms. Multiplexed coculture is crucial to mitigate batch effects when studying the genetic effects of disease-causing variants in differentiated iPSCs or organoids, and demultiplexing at the single-cell level can be conveniently achieved by assessing natural genetic barcodes. Here, to enable cost-efficient time-series experimental designs via multiplexed bulk and single-cell RNA-seq of hybrids, we introduce a computational method in our Vireo Suite, Vireo-bulk, to effectively deconvolve pooled bulk RNA-seq data by genotype reference, and thereby quantify donor abundance over the course of differentiation and identify differentially expressed genes among donors. Furthermore, with multiplexed scRNA-seq and bulk RNA-seq, we demonstrate the usefulness and necessity of a pooled design to reveal donor iPSC line heterogeneity during macrophage cell differentiation and to model rare WT1 mutation-driven kidney disease with chimeric organoids. Our work provides an experimental and analytic pipeline for dissecting disease mechanisms with chimeric organoids.

## Abstract (web summary)

IPSC-derived organoids model diseases. Multiplexed coculture and demultiplexing natural genetic barcodes aid in studying genetic effects. Here, authors introduce Vireo-bulk to deconvolve bulk RNA-seq data, quantify donor abundance and identify differentially expressed genes.

## Introduction

Organoids are important tools for disease modeling and mechanistic studies. They can be derived from adult tissues or differentiated from pluripotent stem cells such as iPSCs^1^. One of the key confounders for applying organoids in disease modeling is technical variability^2^. Reproducibility research with large-scale validation has revealed that experimental differences exist not only across protocols but also between batches and cell lines^3–5^. The effect of technical bias on reproducibility varies by condition, especially in disease modeling, in which the aim is to establish a system to study the effects of genetic differences via organoids. For instance, in disease modeling, organoids may be derived from patient iPSCs, healthy controls, or genetically modified isogenic cell lines. The states of these different cell lines vary subtly, and technical differences among batches and vials amplify the error.

To control for this issue in single-cell RNA-seq results, various computational methods have been introduced for post-correction of batch effects in sequencing data. The basic premise of the batch effect correction algorithm is to assume that a static artifact exists and should be removed to reveal the true biological difference. However, there is a risk that real differences between compared samples might be masked by overcorrection^6^. In organoid-based disease modeling, the difference between samples is emphasized to indicate the gain or loss of function of disease-causing factors; thus, a superior experimental pipeline is needed to eliminate these computational risks.

Multiplexed design is one way to mark different cell sources and mitigate batch effects caused by mixing and coculturing compared cell lines throughout the differentiation process or upon certain treatments^7^. With gene expression data from single-cell RNA-seq as a phenotypic readout, researchers can obtain natural genetic variant information from RNA-seq and leverage SNPs to deconvolve the sequencing reads and assign donor barcodes to single cells by using genotyping-based approaches such as Demuxlet, or reference-free methods such as Vireo and Souporcell^8,9^. However, in time series experiments such as those investigating organoid development, sequencing at single-cell resolution is not sufficiently economically feasible for sequencing all batches with dense sampling at each time point. In differentiation experiments, we aimed to investigate both the differentiated intermediate cells and the final products. It is more cost-efficient to combine single-cell and bulk RNA sequencing in this setting, for instance, performing bulk RNA-seq for multiple time points to dissect differentiation dynamics such as donor proportion change and identifying differentially expressed genes and performing single-cell RNA-seq for final differentiated organoids to acquire a high-resolution atlas for specific cell type profiles. However, no available computational method has been tailored to deconvolving donor abundance and detecting differentially expressed genes in such hybrid multiplexed settings.

Here, we introduced a computation method, Vireo-bulk, that can effectively deconvolve bulk RNA-seq data from multiplexed experimental designs and demonstrated the effectiveness of a combination scRNA-seq and bulk RNA-seq strategy for studying time-series differentiation dynamics of cell proportions and individual gene expression. By applying these methods to studies of iPSC-to-macrophage differentiation and iPSC-to-kidney organoid differentiation, we illustrated the phenomenon of iPSC line heterogeneity and revealed the mechanisms of nephric syndrome caused by a *WT1* mutation.

## Results

### The Vireo suite enables a hybrid time-series strategy for multiplexed experiments to reveal fine-grained organoid differentiation

**Fig. 1:**
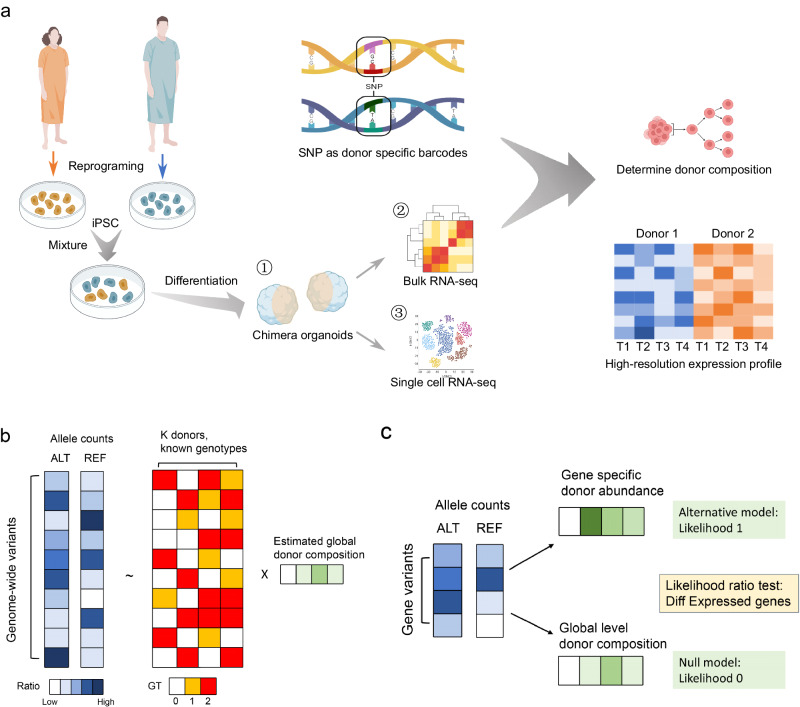
Vireo-bulk: demultiplexing bulk RNA-seq data via natural genetic barcodes. **a** A schematic illustration of the experimental pipeline as applied to chimeric organoids. **b** The computational model Vireo-bulk for demultiplexing mixed donors in bulk RNA-Seq by using expressed allelic reads and the known genotypes. **c** The illustration of detecting differentially expressed genes by a likelihood ratio test. It compares the null model that the donors have the same expression by using the global donor abundance to fit the data, and the alternative model that donors have different abundance by fitting a gene-specific allelic abundance. Image created with BioRender.com and inkscape, with permission.

Multiplexed experimental design with cocultivation is essential to mitigate batch effects when investigating how disease-related genotypes influence phenotypes at the molecular level, especially during organoid differentiation. With single-cell sequencing, these pooled cells can be effectively deconvolved to the donor of origin by leveraging genetic variants as natural barcodes and study the phenotype at the molecular level, such as pluripotency and proliferation^10^. To address the limitations of high cost or low temporal resolution of experiments relying exclusively on scRNA-seq, we introduced a hybrid time-series sequencing strategy by combining both scRNA-seq and bulk RNA-seq at different time points, all in multiplexed settings (Fig. 1a). Therefore, within a lower input of resource, the heterogeneous cell population can be dissected with scRNA-seq, while the high-resolution organoid differentiation dynamics are revealed by bulk RNA-seq.

Despite the success in demultiplexing scRNA-seq, less attention has been given to pooled bulk RNA-seq data. To fill this gap, we extend our Vireo suite by introducing Vireo-bulk, a statistical model that can accurately estimate the donor composition in a multiplexed bulk RNA-seq sample. Briefly, Vireo-bulk uses the genotypes of each donor (e.g., probed by SNP arrays or NGS sequencing; Methods) and models the expressed allele counts (reference or alternative alleles) in the pooled bulk RNA-seq as a result of the donor-specific allelic expression weighted by the unknown proportion of cells in each donor (Fig. 1b; Methods).

Briefly, this model predefined allele frequencies of alternative allele for AA, AB, BB genotypes (e.g., [0.01, 0.5, 0.99]) or automatically learn an adaptive allele frequency vector by default. Admittedly, the pooled allelic proportion is a combined effect of both the number of cells of each donor and the relative expression levels of the genes averaged across all SNPs. However, by considering many SNPs from expressed genes across the whole genome, the overall relative expression levels can be kept comparable among donors on average; hence, the estimated reads composition closely reflects the number of cells in each donor and can be considered to indicate global donor abundance. This model aims to estimate the donor proportion in the pooled RNA-seq sample; therefore, we introduce an Expectation-Maximization (EM) algorithm to obtain a maximum likelihood estimate (see “Methods” and Supp. Algorithm 1).

Furthermore, we can zoom into one gene (or certain gene set) and use only the sequenced SNPs to estimate the abundance of a particular gene (set), which, as mentioned above, is a product of both the genuine donor abundance and the corresponding gene expression level. Therefore, differentially expressed genes (DEGs) between donors can be detected by performing a likelihood ratio test that uses either donor-level abundance (H0 null model: all donors have the same expression) or gene-level abundance (H1 alternative model: donors have different expression causing deviant allelic proportion; Fig. 1c and Methods). Compared to conventional DEG analysis, this multiplexed design offers benefits on both the technical side, by eliminating the batch effects from library preparation to different sequencing batches, and the biological side, by reducing the biological variability through cocultivation.

Taken together with our Vireo-bulk method implemented in the Vireo suite, researchers can seamlessly demultiplex both scRNA-seq and bulk RNA-seq data, making it possible to perform a hybrid time-series experiment. Therefore, we can both minimize batch effects through multiplexing and enable cost-efficient time-series experiments through this hybrid sequencing strategy.

### Evaluation of Vireo-bulk for demultiplexing bulk RNA-seq

**Fig. 2:**
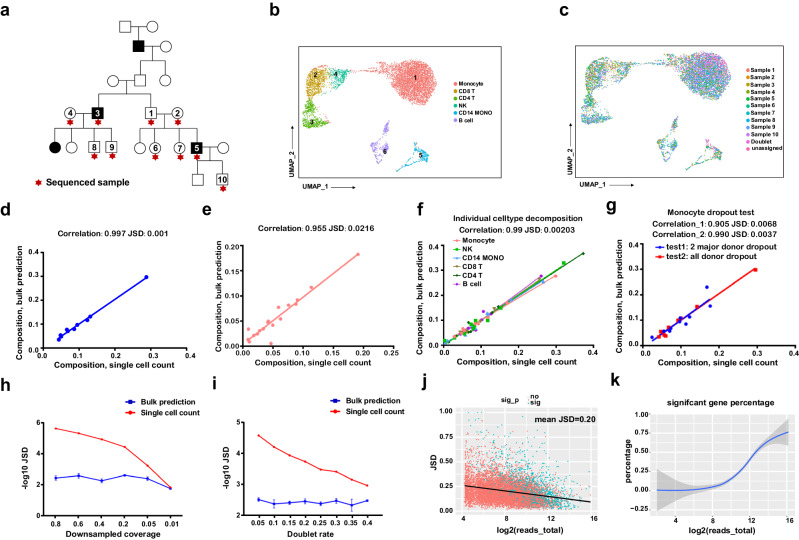
Experimental and synthetic datasets validate the high accuracy of Vireo-bulk method. **a** The pedigree diagram genogram of a family with epilepsy: black represents individuals who are diagnosed with epilepsy symptoms; individuals marked with stars were used for multiplexed scRNA-seq experiment design with genotyped by WGS data as references. **b** The UMAP plot for 10-donor pooled PBMC single-cell transcriptomes clustered by cell type. **c** UMAP plot for single-cell RNA-seq data demultiplexed by the Vireo method with donor information. **d**–**g** Scatter plots of accuracy validation experiments for Vireo-bulk donor composition prediction, where dots present the donor composition in certain donors, with y-axis for bulk RNA-seq predicted by Vireo-bulk versus *x-axis* for counting from single-cell RNA-seq (*x-axis*; treated as ground truth). JSD (Jensen–Shannon divergence) and Pearson correlation were shown in the title. Experiments include accuracy validation both by using all cell types in a 10-donor pool in the above PBMC scRNA-seq data (**d**) and an 18-donor pool from a published dataset on neuron differentiation (**e**), and by synthetically using subsets of cell types in the 10-donor pool with one individual cell type at a time (**f**) and dropping monocyte in all donors or in the two major donors (**g**). **h**, **i** Effects of technical factors on demultiplexing in both bulk and single-cell data, where the *x-axis* is sequencing coverage (**h**) and doublet rate (**i**) and the *y-axis* is -log10(JSD) of donor compositional abundance between the synthetic setting and original setting (treated as ground truth). The error bar denotes the standard deviation (SD) of 3 reruns (technique repeats) of the reads subsampling. (**j**) A scatter plot at the gene level for Vireo-bulk prediction. Each dot represents the JSD and total reads of one gene in all donors between bulk prediction and single-cell reference (treated as pseudo-bulk). Genes are colored in blue if there is a significant change between donors detected Vireo-bulk (*p* < 0.05, two-sided likelihood ratio test) otherwise, red. (**k**) The proportion of significant DE genes varies with the log2(total reads). The DEGs are shown in (**j**). Source data are provided as a Source Data file.

To evaluate the accuracy of Vireo-bulk for estimating donor abundance, we performed accuracy validation experiments with published and in-house experimental datasets and various synthetic datasets. First, we performed multiplexed scRNA-seq on PBMCs from 10 donors (Fig. 2a) from a family affected by epilepsy symptoms. In total, 7,247 cells were obtained with the 10X Genomics scRNA-seq platform and clustered into 6 major immune cell types by using a standard analysis pipeline and annotated with known cell type markers (Fig. 2b and Supplementary Fig. 1a; Methods). We further leveraged Vireo to assign these single cells to the 10 pooled donors by their genotypes probed by whole-genome sequencing (Fig. 2c and Supplementary Fig. 1b,1c).

Then, we used this sample to evaluate the performance of Vireo-bulk by treating it as pseudo-bulk RNA-seq data and keeping only the UMI-tagged reads to achieve precise transcript counting. We found that the donor abundance estimated by Vireo-bulk is perfectly matched with the cell numbers obtained at single-cell resolution (R^2^ = 0.997, Fig. 2d). Similar high consistency was also observed when performing the same analysis on an even larger donor pool (*n* = 18) where iPS cells were differentiated toward neurons (Fig. 2e)^11^. To test whether the accuracy of Vireo-bulk demultiplexing will be influenced by cell types and their composition, we further performed synthetic analyses by keeping or removing a certain cell type from the PBMC scRNA-seq data that was used in Fig. 2b-c. Specifically, we first extracted the reads in scRNA data for each cell type and performed Vireo-bulk for demultiplexing the donor mixture; we found the correlation was perfectly high for all cell types (R^2^ = 0.993, Fig. 2f). Similarly high performance was also observed when dropping one cell type (monocytes) for all donors (Fig. 2g; test 2). Furthermore, we examined the impact of cell type composition change between donors by dropping monocyte on two major donors. The correlation remains high (R^2^ = 0.905, Fig. 2g; test 1) with a slight decrease in this extreme scenario compared to the regular setting, suggesting the robustness of our model.

Furthermore, this high accuracy is robustly retained in Vireo-bulk even when down sampling the sequencing coverage to as low as 1% (equivalent to the typical bulk RNA-Seq sample), while single-cell-level analysis generally requires high coverage to achieve both good-quality cells and reasonable assignability (Fig. 2h, Supplementary Fig. 1d). Additionally, we assessed the effects of the doublet rate on the donor abundance estimation by manually synthesizing 5% to 40% doublets. Unsurprisingly, we found that the scRNA-seq-based estimation suffers remarkably when doublet rates are high, whereas bulk RNA-seq-based estimation is largely resistant to doublets (Fig. 2i).

In addition to deconvolving the donor proportions in bulk RNA-seq, by focusing on gene-specific SNPs, the same Vireo-bulk model can also be applied to quantify the expression level of each donor for a certain gene (set) and consequently identify differentially expressed genes among donors. In a pseudo-bulk manner, Vireo-bulk identified 9,365 genes in the PBMC sample on the basis of SNPs to predict the associated donor abundance, and we used the likelihood ratio test to evaluate the significance of whether these genes are differentially expressed across the donors. Similar to the case for donor proportions, for a given gene (set) in the Vireo-bulk algorithm, we took the total reads assigned to donors as the prediction. For each gene we sequenced, we were able not only to calculate the Jensen–Shannon divergence (JSD) between the true donor abundance vector and the predicted vector but also to perform a likelihood ratio test to determine whether the gene-level expression abundances significantly deviate from the global donor proportions (Fig. 2j, k). Generally, genes with higher total reads, due to higher expression or the presence of more SNPs, produced more accurate gene-level quantitation (i.e., lower JSD values; Fig. 2j). When there were more than 32 reads, the gene-level quantification was reasonably accurate (55.3%, 5179 genes with JSD < 0.2; Fig. 2j). Interestingly, the statistical power of detecting genes with differential expression between donors also has a positive correlation with the total reads covering a gene (Fig. 2k).

### Viero-bulk reveals cell line-specific differential dynamics of multiplexed iPSC differentiated toward macrophages

**Fig. 3:**
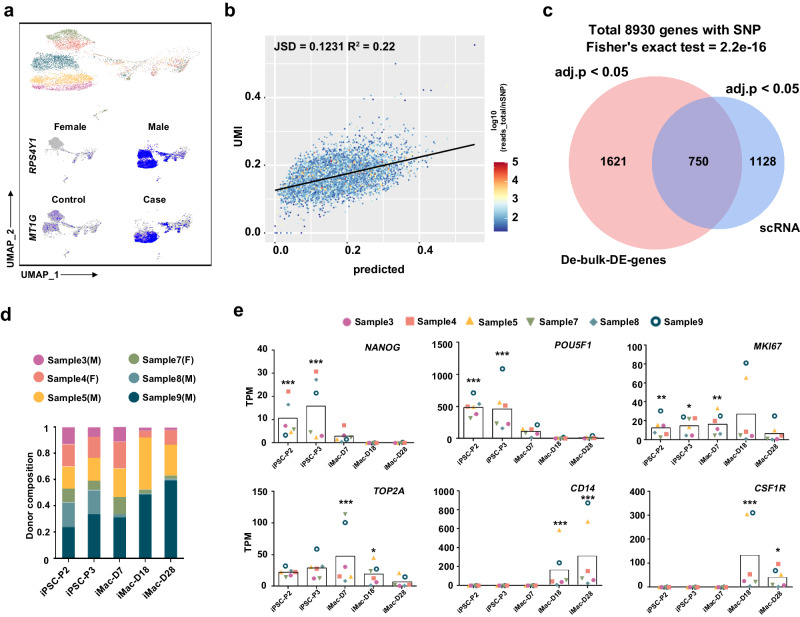
Viero-bulk reveals cell line-specific differential dynamics of multiplexed iPSC differentiated toward macrophages. **a** A UMAP plot of single-cell demultiplexed results by Vireo for 6 pooled donor iPSC samples. Specific sex or disease-related gene expression in demultiplexed single-cell RNA-seq results. Cells are grouped by different donor labels, such as conditions. **b** A scatter plot at the gene level for Vireo-bulk prediction. Each dot represents one gene in one donor’s prediction (*x*-axis) vs UMI counting at single-cell level as ground truth (*y*-axis). **c** A Venn diagram for variable genes detected by Vireo’s pseudo-bulk prediction and variance analysis in single-cell results^32^. Genes with effective SNPs were counted, the adj.p is the cutoff for DE gene selection, and the P value of the Venn plot overlap was calculated by the two-sided Fisher exact test. **d** Dynamic donor composition in the process of differentiation analyzed from pooled bulk-RNA seq demultiplexed by Vireo-bulk. iPSC-P2: iPSCs at passage #2 or iPSCs at the 7th day before differentiation, iPSC-P3: iPSCs at passage #3 or iPSCs immediately before differentiation. **e** Donor-specific gene expression changes in marker genes during differentiation demultiplexed by Vireo-bulk (3 repeats for each pooled sample). The box indicates the average expression level of total pooled samples. The exact P values are calculated by the likelihood ratio test (one side) and provided in the Source Data, which indicates the reliability of the demultiplexing results. Source data are provided as a Source Data file.

After validating the performance on synthetic data, we next applied our hybrid strategy to a real 2D differentiation case. Six isolated PBMC samples collected from the above-described family with epilepsy were reprogrammed to iPSCs, mixed in the same pool for passaging, and induced to differentiate toward a macrophage fate with a stepwise induction protocol^12^-. To study the differential dynamics of the individual cell lines, we performed single-cell RNA-seq with the initial mixture of iPSCs. We found that the donor label presents the major proportion of the variability, especially for sex and disease conditions, which are further confirmed by the distinct expression levels of sex- or disease-related genes, e.g., *RPS4Y1* and *MT1G*, and numerous genes with high proportion of variance explained by sex or disease (Fig. 3a, Supplementary Fig. 2a–f).

By leveraging the pooled single-cell data (iPSC-P2) as a pseudo-bulk sample, we further examined the effectiveness of Vireo-bulk by comparing it to its single-cell level quantification. we also evaluated the gene-level donor abundance that reflects the gene expression level on top of donor abundance and found a reasonable correlation (Pearson’s R^2 = 0.22 for 8930 genes with >= 32 reads; Fig. 3b). Of note, the gene-level quantification is challenging as the number of SNPs is relatively small for a single gene. Third, we also evidenced that Vireo-bulk has a reasonably high concordance in detecting differentially expressed genes between donors compared to the single-cell manner. For 8,930 genes with >=32 total reads, Vireo-bulk detects 2371 DEGs (FDR < 0.05), whereas the single-cell approach in Seurat calls 1,878 DEGs (FDR < 0.05). Interestingly, 750 genes were detected by both methods, indicating a significant overlap (Fig. 3c; p < 10^−7 Fisher’s exact test). Noticeably, this significant overlap between these two strategies is robust to the cutoff used in the DEG analysis (FDR < 0.05 in Fig. 3c or FDR < 0.01 in Supp. Fig. S2c).

To further track the transcriptome differences, we passaged the pooled iPSCs and performed bulk RNA-seq on iPSCs from passage 2 (P2) to passage 3 (P3), as well as samples at day 7, day 18, and day 28 of differentiation. Interestingly, by applying Vireo-bulk to deconvolve the proportion of different donors at each time point, we observed a disproportionate change in individual lines over time (Fig. 3d), even during the passaging process, and ultimately leading to dominant proportions of samples 9, 5 and 4 in the final population.

To identify the possible reasons for disproportionate differentiation, we further demultiplexed the gene expression levels in bulk RNA-seq transcriptome during differentiation. Interestingly, we found meaningful genes with significantly differential expression between donors at the 5-time points, specifically, genes related to pluripotency and proliferation. For instance, the expression of the pluripotency genes *NANOG* and *POU5F1* remained high in iPSCs and decreased as soon as differentiation started for all donors. On the other hand, several lines with high expression levels of proliferation-related genes, such as *MKI67* and the DNA replication-related gene *TOP2A*, also showed dominant proportions (samples 5 and 9). In addition, the expression of macrophage markers (*CD14*, *CSF1R*) increased at days 18 and 28 of differentiation (Fig. 3e). This analysis revealed intrinsic gene expression differences among iPSC lines that could have a significant impact on their proliferation and differentiation potential.

### The hybrid strategy in a kidney organoid disease model

**Fig. 4:**
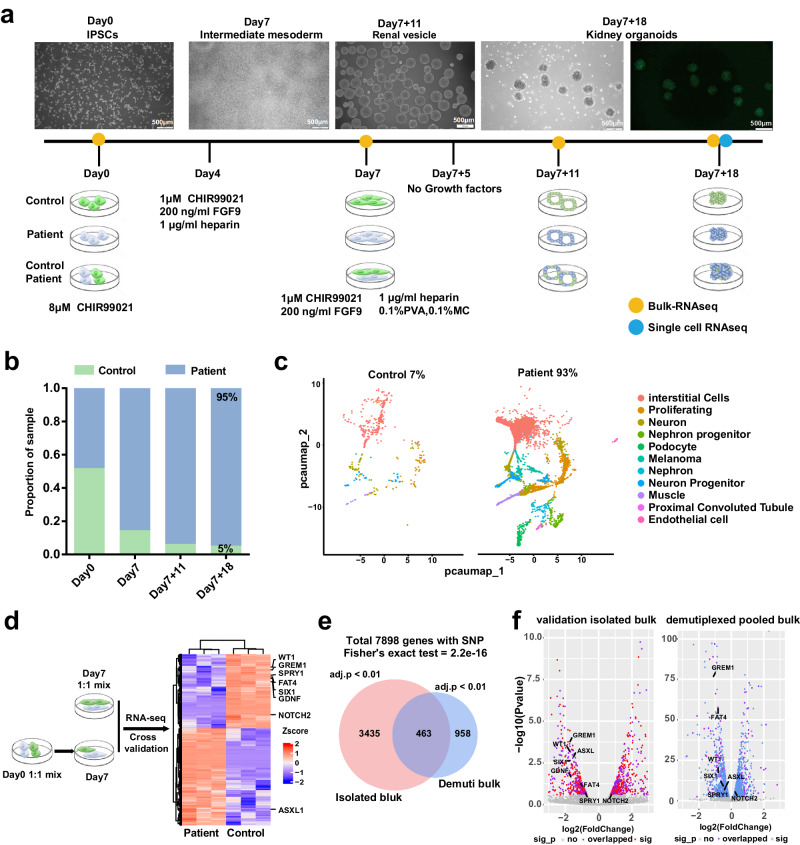
The hybrid strategy in a kidney organoid disease model. **a** The pipeline of the chimeric organoid experiment using the hybrid strategy of single cell and bulk RNA-seq. Representative microscopic pictures and sequencing strategies are illustrated. WT1: iPSCs from a kidney disease donor carrying a *WT1* mutation. Control: iPSCs from a healthy individual without kidney disease. Image created with BioRender.com and inkscape, with permission. **b** Donor composition changes in chimeric organoid differentiation demultiplexed by Vireo-bulk. **c** Single-cell demultiplexed results of chimeric organoids at day 25. **d** Design and results of Vireo-bulk validation experiments. The heatmap shows the pooled bulk demultiplexed results on day 7. Labeled genes are genes considered to be related to the *WT1* gene. Image created with BioRender.com and inkscape, with permission. **e** A Venn plot of differentially expressed genes (DEGs) on day 7 in 1:1 mixed, sequenced, and demultiplexed cells versus day 7 DEGs discovered by DEseq2 in separately sequenced samples. The adj.p is the cutoff of selected genes, and the P value of the Venn plot was calculated by the two-sided Fisher exact test. **f** Volcano plots for isolated sequencing and pooled sequencing DEGs. The value is calculated by DEseq2’s Wald test. Source data are provided as a Source Data file.

To further demonstrate the power of the hybrid strategy, we applied it to a kidney organoid disease model constructed by pooling cells from a healthy donor and a patient donor. The patient was diagnosed with nephrotic syndrome at 13 years old, and the patient had a family history of renal inherited disease. The father and grandmother of the patient were already in End-Stage Renal Disease (ESRD). The sequencing analysis identified heterozygous, single-base-pair WT1 variants at c.1306 A > G (p.R436G, NM_024426.6) (Supplementary Fig. 3a). The periodic-acid silver methenamine–stained sections of kidney biopsy material from the proband demonstrate segmental sclerosis of glomeruli characteristic of FSGS (Supplementary Fig. 3a). We established a kidney organoid differentiation protocol based on previous studies (Fig. 4a)^13^. We performed bulk RNA-seq at multiple time points and scRNA-seq at the final time point of day 25. After demultiplexing the bulk RNA-seq transcriptome data by Vireo-bulk, we discovered that in the mixed sample, the proportion of cells contributed by the *WT1* mutant donor increased rapidly, and consequently, this donor became dominant during differentiation (95%:5%; Fig. 4b). This dominance was also observed from the single-cell counts in the scRNA-seq from the chimeric organoid on day 25 demultiplexed by Vireo (93%:7%; Fig. 4c).

To further evaluate the performance of Vireo-bulk in detecting DEGs, patient and healthy control iPSC lines were separately cultured but pooled with joint sequencing on day 7. Meanwhile, the isolated reference samples were also sequenced separately with bulk RNA-seq, and Vireo-bulk was applied to the mixed sample to identify the DEGs (Fig. 4e, f). The expression of *WT*1-related genes in the mixed and demultiplexed samples showed the same tendency of change as in the separately sequenced samples (Fig. 4d). Moreover, a comparison of the overlapping DEGs from the separated samples (by DESeq2) and the mixed sample (by our Vireo-bulk) showed that the predicted DEGs from the mixed and demultiplexed samples were bona fide DEGs, indicating that our method is capable of effectively capturing DEGs. Among the total 7,898 genes with >=32SNPs analyzed by both strategies, 2717 and 1457 genes were detected as DEGs by isolated bulk sequencing and multiplexed bulk sequencing strategies, respectively, with a significant overlap (718 genes; p < 1e-7; Fig. 4e, f).

### Chimera kidney organoids provide a model for studying genetic diseases

**Fig. 5:**
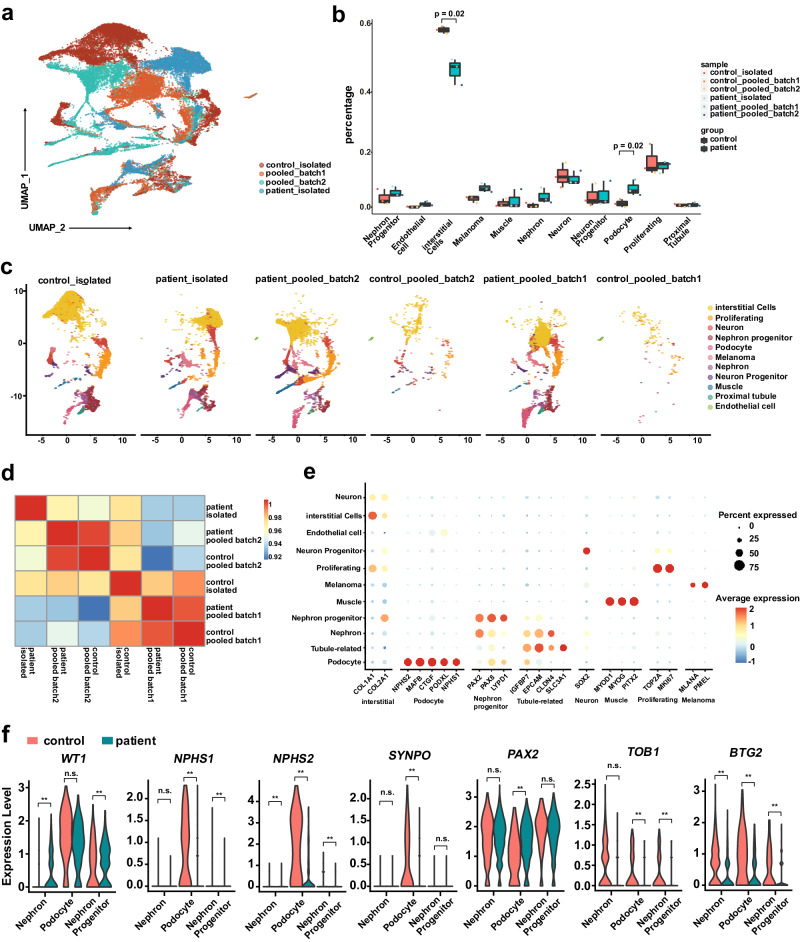
Demultiplexed results of mature chimeric organoids reveal the mechanisms of WT1-mediated kidney disease. **a** A UMAP plot of kidney organoids from 4 batches(30,294 cells). **b** Proportional abundance of different cell types where samples are normalized by total cell count. Standard boxplot was used with dots denoting each batch_samples that are colored as in panel a. In the box plots, the center line represents the median, the box limits indicate the 25th and 75th percentiles and the whiskers extend to the maximum and minimum data points. *P* value is calculated by a one-sided t-test. **c** ScRNA-seq UMAP plots for 4 batches, and cells are colored by batch sample labels). **d** Correlation heatmap analysis of different batch samples; correlation is calculated by different cell type proportions. **e** Dot plots of functional markers of organoids. Marker genes are clustered by cell type reference. **f** Violin plots of *WT1* and downstream marker gene expression in selected cells. Patient samples are shown in green, and control samples are shown in red. *: *P* < 0.05, **: *P* < 0.01; The exact P value is calculated by a one-sided *t*-test and provided in *t*he Source data. Source data are provided as a Source Data file.

Next, we collected mature chimeric organoids and isolated samples to explore whether this model can be used to study kidney genetic disease via a multiplexed strategy. Four batches, specifically, one isolated control, one isolated patient, and two mixed pooled samples, were sequenced at the single-cell level, yielding data for 30,294 effective cells in total after quality control (Methods). Pooled samples were demultiplexed with Vireo and labeled with donor source and batch information. Even though here we did not perform the bulk RNA-seq and apply the Vireo-bulk, the principle of the method should work as well. We observed a significant batch effect across the four different batches in the UMAP cell embeddings (Fig. 5a), even for the same donors in the mixed and isolated batches, which further supports the necessity of cocultivation in the organoid model. Therefore, we applied in silico batch correction to integrate these four batches for joint analysis with Harmony (Supplementary Fig. 3b and c; Methods). Furthermore, we performed clustering and manual cell type annotation. The cell type marker genes supported that the major subgroups of cells exist in the kidney organoids (Fig. 5e). Surprisingly, when comparing the cell type proportions, the samples in the same batch returned a higher Pearson’s correlation (R = 0.993 for batch 1 & R = 0.995 for batch 2) than those from the same donor (R = 0.927 for patient & R = 0.931 for control; Fig. 5d), again suggesting that the culture batch may introduce obvious noise.

Previous research showed that *WT1* mutation leads to dysregulation of podocyte differentiation and proliferation and that these phenotypes are associated with podocyte-related kidney diseases^14–16^. Due to the mutation of *WT1*, the corresponding progenitor cells could exhibit abnormally elevated proliferation, which might cause an increased proportion of podocytes with mutant *WT1* gene expression. In our single-cell sequencing results above, the percentage of podocyte and nephron cells from the *WT1* mutation-carrying patient was significantly higher than that from the healthy control (Fig. 5b, c).

Then, we asked whether we could use the organoid system to reveal the differentially expressed genes caused by the *WT1* mutation to help us to study the disease. We divided the cells into three major groups (Supplementary Fig. 3d), and variance-decomposition analysis showed the potential factors existed in the separated sample leads to gene expression variance. suggests the necessity of pooled analysis and (Supplementary Fig. 3f and 3g). Many *WT1*-related genes showed differential expression in mutants compared to healthy controls (Fig. 5f), and these genes were related to kidney diseases (Supplementary Fig. 3e). Specifically in the podocyte cluster, *WT1* mutation led to a significantly decreased expression of *NPHS1*, *NPHS2,* and *SYNPO*, which maintain the specific cellular structure of podocytes. *WT1* mutation also led to a decrease in proliferation-related gene *TOB1* and *BTG2* expression in the nephron, nephron progenitor, and podocyte cell clusters. Besides, in the podocyte cluster *WT1* mutation also led to an increase in podocyte differentiation-related gene *PAX2* expression, which inhibits podocyte maturation. Further, the immunofluorescence assays also showed that nephron development markers SALL1/PAX8 protein expression level was increased in the patient kidney organoids, and podocyte-related markers WT1/NPHS1 protein expression level was up-regulated in the control kidney organoids (Supplementary Fig. 4a and 4b). These results support that the *WT1* mutation causes abnormalities in podocyte differentiation and proliferation in the FSGS patient^17–19^.

In summary, we showed the chimeric organoids combined with the demultiplexing methods can be applied in studies of disease models.

## Discussion

The iPSC-differentiated organoid is a powerful platform for disease modeling, but its application is hindered by poor batch-to-batch reproducibility in organoid production. Here, we developed a multiplexed batch-free experimental design and a demultiplexing computational pipeline based on both single-cell and bulk RNA sequencing to overcome the above challenges. This method can be used to reveal donor/clone dynamics during iPSC differentiation, identify differentially expressed genes/pathways in chimeric organoids, and establish genotype–phenotype relationships of modeled diseases.

The method can be applied to other chimeric donor contexts, such as allogeneic transplantation. Currently, short tandem repeat (STR) analysis is used to determine chimerism after allogeneic hematopoietic stem cell transplantation (HSCT) for patients with various hematologic malignancies. However, the sensitivity of STR is moderate, as there are only a small number of STRs included in the panel. The method above can be theoretically applied to this context with much higher sensitivity, as the number of donor-specific SNPs or SNP-containing genes is much larger. Along these lines, the method might be applied to emerging “off-the-shelf” or universal allogeneic immune cell therapies to determine donor cell persistence and fraction and evaluate donor–recipient cell interactions.

There are also limitations to our method. First, although genotype information for each donor is not necessary for demultiplexing in single-cell data by Vireo, it is required for Vireo-bulk to deconvolve bulk RNA-seq data. Even though we can obtain SNP-coded genotype information from single-cell RNA-seq or bulk RNA-seq, for better sensitivity in certain experimental models, DNA-based genotype chips or genome sequencing results are still needed for genotyping information. Second, Vireo-bulk detects differentially expressed genes between donors based on allelic reads, which eliminates the requirement of library size normalization in conventional bulk RNA-seq samples. However, as there are fewer SNPs in certain genes, the sensitivity for distinguishing their donor-specific expression might be affected, especially as the number of donors increases. Third, with simulations we demonstrated that our method is relatively robust to the cell type composition change for estimating the donor proportion. On the other hand, for the donor specific gene expression estimation, it is tightly coupled with cell type composition, hence one should interpret the gene expression change with the potential reason of cell type composition change.

## Methods

### Ethics statement

Written informed consent was obtained from the patient, and the study was approved by the Human Subjects Committee of Jinling Hospital, Nanjing University (2021DZGZR-YBB-109). The participants of the experiment of PBMC, iPSC, and iMac: samples 1-4 are from 60-70 years old donors, and samples 5–10 are from 10–40 years old donors. Sample 1/3/5/8/9/10 are male, and sample 2/4/6/7 are female. The participant of the experiment of kidney organoid: *WT1* mutation sample was from a 20–30 years old male donor.

### Vireo-bulk algorithm

In multiplexed bulk RNA-seq samples in which multiple donors are pooled in one experiment, our deconvolution method, Vireo-bulk, aims to estimate the proportions of read counts coming from each of the *K* donors through modeling of the expressed alleles governed by the distinct genotypes of each donor. Here, we consider *N* effective biallelic variants (or SNPs) sequenced that have different genotypes in at least two donors in the pool and sufficient read counts sequenced, for example, at 100 reads or UMIs and >5% from minor alleles, which can be obtained by probing with SNP arrays or high-throughput sequencing platforms (see genotyping section below). For a certain variant *i* in donor *k*, its genotype can be written as ${g}_{i,k,t}\in \{0,\,1\}$, where $t\in \{{{{{\mathrm{0,1,2}}}}}\}$ refers to the category of the genotype for homozygous reference BB, heterozygous AB, and homozygous alternative AA. In theory, the corresponding expressed allele frequency of alternative allele A in genotypes BB, AB, and AA should be ***θ*** = {${\theta }_{0},{\theta }_{1},{\theta }_{2}$}={0, 0.5, 1}, though deviation may occur due to technical noise, e.g., sequencing errors and genotyping errors, or allele-specific expression (ASE) effects. Nonetheless, these parameters ***θ*** = {${\theta }_{0},{\theta }_{1},{\theta }_{2}$}, which are globally shared across all variants and donors, can be estimated adaptively in our model (see below). By taking the known genotypes while ignoring this deviance, the allelic expression of variant *i* for each donor *k* in the mixture can be obtained as follows:

1 $${\mu }_{i,k}={\sum }_{t=0}^{2}\, {g}_{i,k,t}{\theta }_{t},\, t\in \{0,1,2\}$$

Meanwhile, for variant *i* in the multiplex bulk sample, for each A and B allele sequenced, the read count supporting the alternative allele is ${a}_{i}$ and reference allele is ${b}_{i}$ out of the ${d}_{i}={a}_{i}+{b}_{i}$ total reads. For a certain read *j*, the observation of its allele ${r}_{j}\in \{A,B\}$ follows a Bernoulli distribution with genotype specific parameter ${\theta }_{T}$ given the genotype is T (i.e., ${g}_{i,k,T}=1$), as follows:

2 $$\left\{\begin{array}{c}{{{{{\rm{P}}}}}}\left({r}_{j}={{{{{\rm{B}}}}}} | {\theta }_{T}\right)={\theta }_{T}\\ P\left({r}_{j}={{{{{\rm{A}}}}}} | {\theta }_{T}\right)=1-{\theta }_{T}\end{array}\right.$$

Given the donor proportion ${{{{{\boldsymbol{\phi }}}}}}=\{{\phi }_{1},{\phi }_{2}...{\phi }_{K}\}$ with ${\sum }_{{k}{=}{1}}^{{K}}{{\phi }}_{{k}}{=}{1}$. For a specific variant *i*, the likelihood of observing total ${a}_{i}$ reads from the A allele and ${b}_{i}={d}_{i}-{a}_{i}$ reads from the B allele can be expressed as follows:

3 $$\begin{array}{c}{{{{{\rm{p}}}}}}\left({a}_{i},{b}_{i}|{{{{{{\rm{\mu }}}}}}}_{i},{{{{{\rm{\phi }}}}}}\right)={\prod }_{j=1}^{{a}_{i}}{\sum }_{k=1}^{K}P\left({r}_{j}=A | {{{{{{\rm{\mu }}}}}}}_{i},k\right)P\left({I}_{j}=k | {{{{{\rm{\phi }}}}}}\right)\\ \times {\prod }_{j=1}^{{b}_{i}}{\sum }_{k=1}^{K}P\left({r}_{j}=B | {{{{{{\rm{\mu }}}}}}}_{i},k\right)P\left({I}_{j}=k | {{{{{\rm{\phi }}}}}}\right)\\ {=}{\left({{{{{{{\boldsymbol{\mu }}}}}}}}_{{i}}^{{{{{{{\rm{\top }}}}}}}}{{{{{{\boldsymbol{\phi }}}}}}}\right)}^{{{a}}_{{i}}}{\left({\left({{{{{{\bf{1}}}}}}}{-}{{{{{{{\boldsymbol{\mu }}}}}}}}_{{i}}\right)}^{{{{{{{\rm{\top }}}}}}}}{{{{{{\boldsymbol{\phi }}}}}}}\right)}^{{{b}}_{{i}}}\,=\,{\left({{{{{{{\boldsymbol{\mu }}}}}}}}_{{i}}^{{{{{{{\rm{\top }}}}}}}}{{{{{{\boldsymbol{\phi }}}}}}}\right)}^{{{a}}_{{i}}}{\left({1}{-}{{{{{{{\boldsymbol{\mu }}}}}}}}_{{i}}^{{{{{{{\rm{\top }}}}}}}}{{{{{{\boldsymbol{\phi }}}}}}}\right)}^{{{b}}_{{i}}}\end{array}$$

This likelihood is in the same form as the binomial distribution by taking the averaged allele rate across donors ${{{{{{{\boldsymbol{\mu }}}}}}}}_{{i}}^{{{{{{{\rm{\top }}}}}}}}{{{{{{\boldsymbol{\phi }}}}}}}$. With conditional independence, the joint likelihood of $N$ variants can be written by taking their product as follows:

4 $$L\left({{{{{\boldsymbol{\theta }}}}}},{{{{{\boldsymbol{\phi }}}}}}\right)={\prod }_{i=1}^{N}P\left({a}_{i},{b}_{i} | {{{{{{\boldsymbol{\mu }}}}}}}_{i},{{{{{\boldsymbol{\phi }}}}}}\right)={\prod }_{i=1}^{N}{\left({{{{{{\boldsymbol{\mu }}}}}}}_{i}^{{{{{{\rm{\top }}}}}}}{{{{{\boldsymbol{\phi }}}}}}\right)}^{{a}_{i}}{\left(1-{{{{{{\boldsymbol{\mu }}}}}}}_{i}^{{{{{{\rm{\top }}}}}}}{{{{{\boldsymbol{\phi }}}}}}\right)}^{{b}_{i}}$$

By introducing an Expectation-Maximization algorithm (Algorithm 1; below”)we obtain a maximum-likelihood estimation of ${{{{{\boldsymbol{\phi }}}}}}$ and ${{{{{\boldsymbol{\theta }}}}}}$. Alternatively, the genotype-specific allelic expression parameter ${{{{{\boldsymbol{\theta }}}}}}$ can be set to a fixed value, e.g., $\theta=\left\{{\theta }_{0},{\theta }_{1},{\theta }_{2}\right\}=\left\{0.01,\,0.5,\,0.99\right\}$ as default.

### Detection of differential gene expression between donors with Vireo-bulk

In addition to quantifying the donor abundance by using genome-wide SNPs, Vireo-bulk can also be used to focus on a certain gene *g* (or a gene set with similar functions) and its SNPs. Therefore, the estimated value from this subset of SNPs reflects the product of the number of cells from each donor and their mean expression. If the expression levels of gene *g* are highly similar between donors, the estimated ${{{{{{\boldsymbol{\phi }}}}}}}_{g}$ is expected to be close to the global donor abundance. In other words, a ${{{{{{\boldsymbol{\phi }}}}}}}_{g}$ that is substantially different from the global donor abundance ${{{{{\boldsymbol{\phi }}}}}}$ implies that significant differential expression exists in at least one donor.

Therefore, we introduced a likelihood ratio test to detect the differential gene expression between donors. Specifically, we compare the likelihood of observing the read counts for a certain gene $g$ when using the global donor abundance ${{{{{\boldsymbol{\phi }}}}}}$ (null hypothesis with no differential expression) and when using the specific estimated gene abundance ${{{{{{\boldsymbol{\phi }}}}}}}_{g}$ (alternative hypothesis with differential expression) as follows:

5 $${H}_{0}:{L}_{0}\left({a}_{g} | {d}_{g},{{{{{\boldsymbol{\phi }}}}}},{{{{{\boldsymbol{\theta }}}}}}\right)=P\left({a}_{g} | {d}_{g},{{{{{\boldsymbol{\phi }}}}}},{{{{{\boldsymbol{\theta }}}}}}\right)={\prod }_{i=1}^{{N}_{g}}p\left({a}_{i} | {d}_{i},{{{{{{\boldsymbol{\mu }}}}}}}_{i},{{{{{\boldsymbol{\phi }}}}}}\right)$$

6 $${H}_{1}:{L}_{1}\left({a}_{g} | {d}_{g},{{{{{\boldsymbol{\phi }}}}}},{{{{{\boldsymbol{\theta }}}}}}\right)=P\left({a}_{g} | {d}_{g},{{{{{\boldsymbol{\phi }}}}}},{{{{{\boldsymbol{\theta }}}}}}\right)={\prod }_{i=1}^{{N}_{g}}p\left({a}_{i} | {d}_{i},{{{{{{\boldsymbol{\mu }}}}}}}_{i},{{{{{{\boldsymbol{\phi }}}}}}}_{g}\right)$$

Then, we calculate the likelihood ratio test statistic ${{{{{\rm{\lambda }}}}}}=-2\log \left({L}_{0}/{L}_{1}\right)$ and assume that it follows a Chi-square distribution with K-1 degrees of freedom to obtain a one-tailed *p* value.

Generally, gene-level abundance and differential expression tests require a sufficient number of SNPs with distinct genotypes between donors. In this study, we focused on 4000 genes covering 20,000 SNPs in total.

### Genotyping calling

Different samples in different experiments are genotyped via different pipelines. For epilepsy family samples, raw WGS results were aligned to GRCh38 (Ensembl 93) via Sentieon ® bwa and joint calling was performed by the standard Sentieon® pipeline^20^. The genotype calling result was validated with the same parameter in the GATK pipeline^21^. For organoid donors, their genotype reference was called according to the RNA-seq results. For day 0 to day 14 organoid samples, isolated individual bulk RNA-seq samples were aligned to GRCh38 by HISAT 2.0, and bam files were processed by cellSNP-lite bulk mode to call donor-specific genotypes^22^. For samples collected after day 14, genotypes are called from isolated single-cell transcriptomes. Processed bam files given by the cellranger default pipeline were piled up via cellSNP-lite bulk mode and used as the reference for samples after day 14^23^.

### RNA-seq data processing and analysis

Raw RNA-seq reads generated by Illumina in fastq format were first trimmed with the Trimmomatic tool and then aligned to the human genome (hg38 from GENCODE) by HISAT2 with default parameters^24^. Multimapped reads and PCR duplicates were masked for subsequent quantification and genotyping with RepeatMask. After the gene expression count was qualified by FeatureCounts, the edgeR package was used for count normalization and differential expression analysis. The FDR cutoff for DEGs was 0.1^25,26^.

For the kidney organoid data, sample-separated bulk RNA-seq datasets are available; hence, the aligned bam files were used for genotyping through FreeBayes with the parameter -min-alternate-fraction 0.2^27^. Then, the generated VCF file was filtered with -minQ 30 by BCFtools.

### Single-cell expression matrix generation and stimulating data preprocessing

For reads produced by the 10X-Chromium V3 protocol, including both repeats and coding genes, single-cell RNA-seq was generated by 10X Genomics Chromium (chemical v2). The sequencing reads in fastq format were trimmed and then mapped to the hg38 genome index by the default cell ranger-3.0.2 pipeline. We used the cells called by cell ranger in the default setting. Bam files were genotyped at given known SNPs (FreeBayes called before) by CellSNP-lite 1.2.1 and demultiplexed by Vireo 0.2.3. Only cell barcodes predicted as singlets by Vireo were kept for generating simulated data by subsampling.

### Analysis of scRNA-seq data

After inputting the UMI count sparse matrix from the cell ranger 3.0.2., DGE was normalized by log2(TPM)^28^. The Seurat R package (v4.0) was used for downstream analysis of single-cell transcriptome data from the cell ranger cell-by-gene UMI count matrix^29^. The top 2000 variable genes in the cleaned DGE were detected via VST methods by the FindVariableFeatures function. Then, the top 20 PCs were selected using the Jackstraw function, and their coordinates were used for uniform manifold approximation (UMAP) to generate low-dimensional cell embedding and SNN clustering (resolution=0.4).

### Single-cell organoid atlas mapping and batch effect correction

The kidney organoid atlas was preprocessed by the Seurat R package (v4.0) with annotated cell type metadata. Query data were mapped to the atlas through TransferData (aweight.reduction = “cca”, dims = 1:30) after anchored reference FindTransferAnchors (1:30, reference.assay, normalization.method = “LogNormalize”, reduction = “cca”). The batch effect was calculated by the Jensen–Shannon divergence between proportions of cell types. For batch effect removal methods in the embedding plot, Harmony with default parameters was used to generate the dimensionally reduced matrix.

### Reprogramming of donor PBMCs to iPSCs

Fresh whole blood was obtained from consenting donors, and then the PBMC Isolation Kit (Solarbio P8610) was used to isolate PBMCs from the samples. The PBMCs were cultured in H3000 (STEMCELL Technologies, Catalog # 100-0073) with CC100 (STEMCELL Technologies, Catalog # 02690). Subsequently, these PBMCs were electroporated with OriP/EBNA-1-based episomal plasmids expressing the reprogramming factors OCT3/4, SOX2, KLF-4, L-MYC, and LIN28^30^.

### Generation of kidney organoids

iPSCs were induced to differentiate toward the primitive streak by treating cells with 7 μM CHIR99021(STEMCELL Technologies, Catalog # 72052) in TeSR-E6(STEMCELL Technologies, Catalog # 05946) medium for 4 days. Next, 200 ng/ml FGF9(MCE, Catalog # HY-P7177), 1 μg/ml heparin(STEMCELL Technologies, Catalog # 07980) and 1 μM CHIR99021 were added to induce the iPSCs to differentiate toward intermediate mesoderm (IM) for 3 days. The IM cells were digested into single cells, resuspended in 200 ng/ml FGF9, 1 μg/ml heparin, 1 μM CHIR99021, 0.1% PVA, 0.1% MC, and 10 μM ROCK inhibitor(STEMCELL Technologies, Catalog # 72308) medium, and cultured in a horizontal shaker. After 24 hours, the ROCK inhibitor was removed from the medium. On the next five days, all cytokines were removed and maintained in TeSR-E6 medium. Organoids were spontaneously formed in the following 13 days.

### Immunofluorescence staining

Kidney organoids were fixed with 2% PFA for 20 min and incubated with primary antibodies overnight at 4 °C. The kidney organoids were then washed five times with PBS and incubated with secondary antibodies with fluorescent labels. After staining, the kidney organoids were dehydrated using a 25%, 50%, 75%, and 100% methanol series for 5 min, followed by clearing using benzyl alcohol and benzyl benzoate (BABB, 1:2 ratio). The clear kidney organoids were mounted on a glass-bottom dish (NEST Corporation). The stained cells and kidney organoids were observed via confocal microscopy (Nikon). The primary antibodies: WT1 (1:100, abcam, cat. no. ab89901), SALL1 (1:100, Thermo, cat. no. PA5-62057), PAX8 (1:100, Proteintech, cat. no. 10336-1-AP), NPHS1 (1:100, R&D System, cat. no. AF4269). The secondary antibodies: Donkey Anti-Rabbit IgG H&L Alexa Fluor® 488(1:200, abcam,ab150073), Donkey Anti-Rabbit IgG H&L Alexa Fluor® 647(1:200, abcam,ab150075), Donkey Anti-Rabbit IgG H&L Alexa Fluor® 568(1:200, abcam, ab175470), Donkey Anti-Sheep IgG H&L Alexa Fluor® 647 (1:200, abcam, ab150179), Donkey Anti-Sheep IgG H&L Alexa Fluor® 488(1:200, abcam, ab150177).

### Statistics & Reproducibility

Statistical analyses were conducted utilizing R statistical software (version 3.6.1) and Python (version 3.10). In Vireo-bulk, Differentially Expressed Genes (DEGs) between donors were identified through the execution of a likelihood ratio test. For the significance test in the Venn diagram, Fisher’s exact test was employed. For isolated sample’s DEGs significance testing, p-values were calculated using the Wald test from the DEseq2 package. For significance testing in the violin plot, a one-sided t-test was performed. All correlations were assessed using Spearman’s rank correlation coefficient. A p-value of less than 0.05 was considered statistically significant in all analyses.

No statistical method was used to predetermine the sample size. No data were excluded from the analysis. The experiments were not randomized, and the investigators were not blinded to allocation during experiments and outcome assessment.

To ensure reproducibility, all methods and experimental protocols were meticulously described in the Methods section. The data and code utilized for the analyses are readily available in the Supplementary Information and have also been deposited in a public repository.

### Reporting summary

Further information on research design is available in the Nature Portfolio Reporting Summary linked to this article.

### Supplementary information

Supplementary Information

Reporting Summary

### Source data

Source data

## Data Availability

All relevant data supporting the key findings of this study are available within the article and its Supplementary Information files. The multiplexed neuron differentiation data (18-donor pool; used in our Fig. 2e-g) was previously published and available on EGA under the dataset EGAD00001006157. All datasets generated by this study (Macrophage differentiation scRNA-seq, chimera kidney organoid bulk RNA-Seq and scRNA-seq datasets) are publicly available on National Genomics Data Center (NGDC) with project number PRJCA024329. Source data are provided in this paper.

## Code availability

The source codes are publicly available at https://github.com/chengarthur/Vireobulk_analysis, and the documentation can be found at: https://vireobulk-analysis.readthedocs.io. DOI for the GitHub repository, 10.5281/zenodo.10823711^31^.
