# Author Correction: Multiplexed bulk and single-cell RNA-seq hybrid enables cost-efficient disease modeling with chimeric organoids

**DOI:** 10.1038/s41467-024-48984-w

**Published:** 2024-06-05

**Authors:** Chen Cheng, Gang Wang, Yuqing Zhu, Hangdi Wu, Li Zhang, Zhihong Liu, Yuanhua Huang, Jin Zhang

**Affiliations:** 1 Center for Translational Stem Cell Biology, Hong Kong Science and Technology Park, Hong Kong SAR, China; 2 Liangzhu Laboratory, Zhejiang University School of Medicine, Hangzhou, China; 3 School of Biomedical Sciences, Li Ka Shing Faculty of Medicine, The University of Hong Kong, Pokfulam, Hong Kong SAR, China; 4 Center for Stem Cell and Regenerative Medicine, Department of Basic Medical Sciences, and Bone Marrow for Transplantation Center of the First Affiliated Hospital, Zhejiang University, Hangzhou, China; 5 National Clinical Research Center of Kidney Diseases, Jinling Hospital, Nanjing University School of Medicine, Nanjing, China; 6 Department of Basic Medical Sciences, Zhejiang University School of Medicine, Hangzhou, China; 7 Center for Stem Cell and Translational Medicine, School of Life Sciences, Anhui University, Hefei, Anhui China; 8 Department of Statistics and Actuarial Science, The University of Hong Kong, Pokfulam, Hong Kong SAR, China; 9 Center of Gene/Cell Engineering and Genome Medicine of Zhejiang Province, Hangzhou, China

**Keywords:** Computational models, Kidney diseases, RNA sequencing, Lab-on-a-chip, Induced pluripotent stem cells

Correction to: *Nature Communications* 10.1038/s41467-024-48282-5, published online 10 May 2024

In this article, Chen Cheng and Gang Wang should have been denoted as equally contributing authors. The original article has been corrected.

