# Supplementary material for: Multiplexed bulk and single-cell RNA-seq hybrid enables cost-efficient disease modeling with chimeric organoids
Source: Nat Commun. 2024 May 10;15:3946. doi: 10.1038/s41467-024-48282-5 (PMC11087505; doi:10.1038/s41467-024-48282-5)
Supplement: Supplementary file 1 — Supplementary Information [file 41467_2024_48282_MOESM1_ESM.pdf]

Multiplexed bulk and single-cell RNA-seq hybrid enables cost-efficient disease modeling with chimeric organoids

Chen Cheng et al

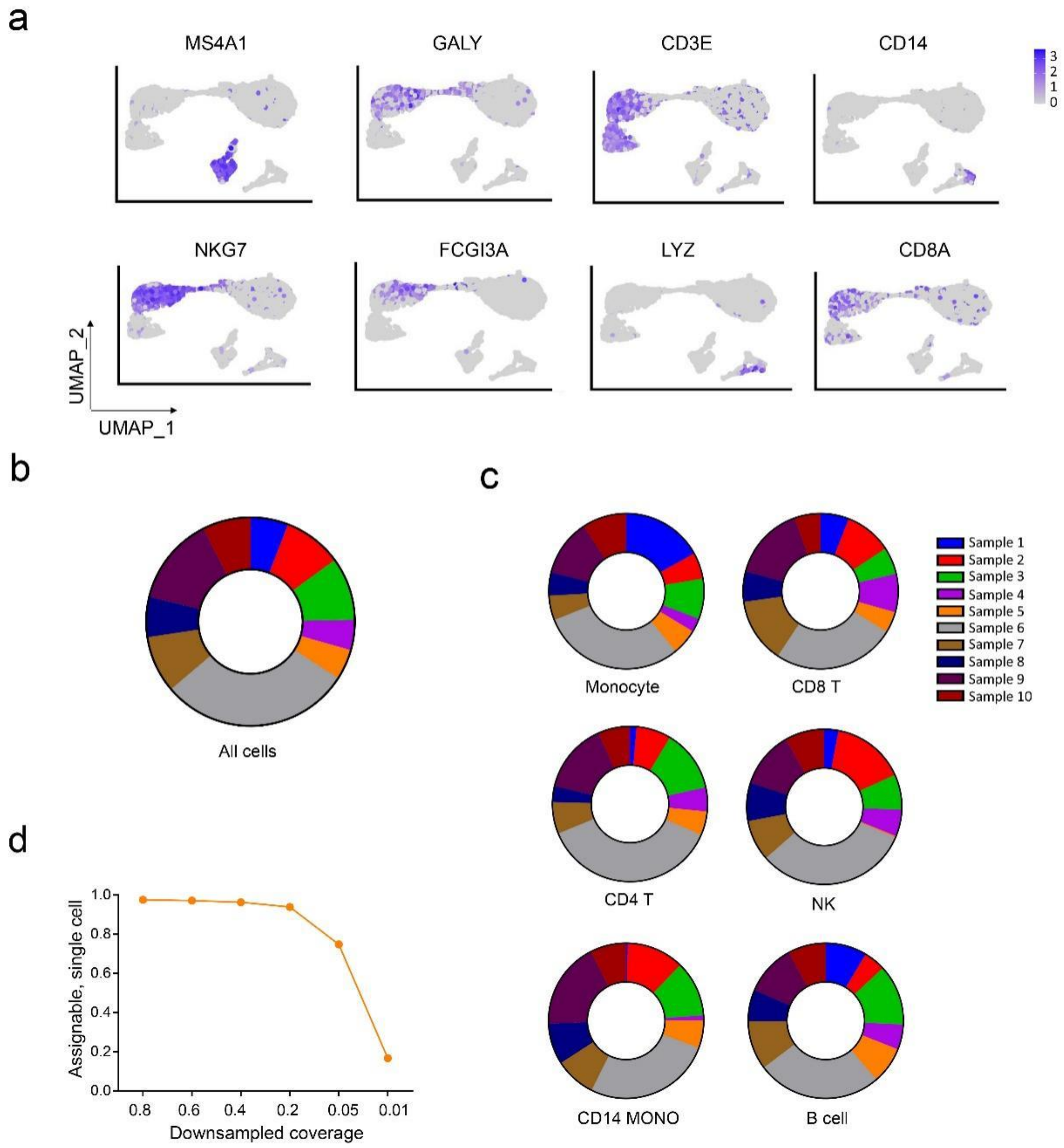

Supplementary Fig. 1. Cluster information and cluster markers for 10-donor-mixed PBMCs. (a) PBMC marker expression in 10-donor multiplexed single-cell RNA-seq data. (b) Percentage of each donor's cells in pooled PBMC samples. (c) Percentage of each donor's cells in each specific cell type. (d) The proportion of assignable cells in single-cell RNA-seq demultiplexing varies during down sampling. Source data are provided as a Source Data file.

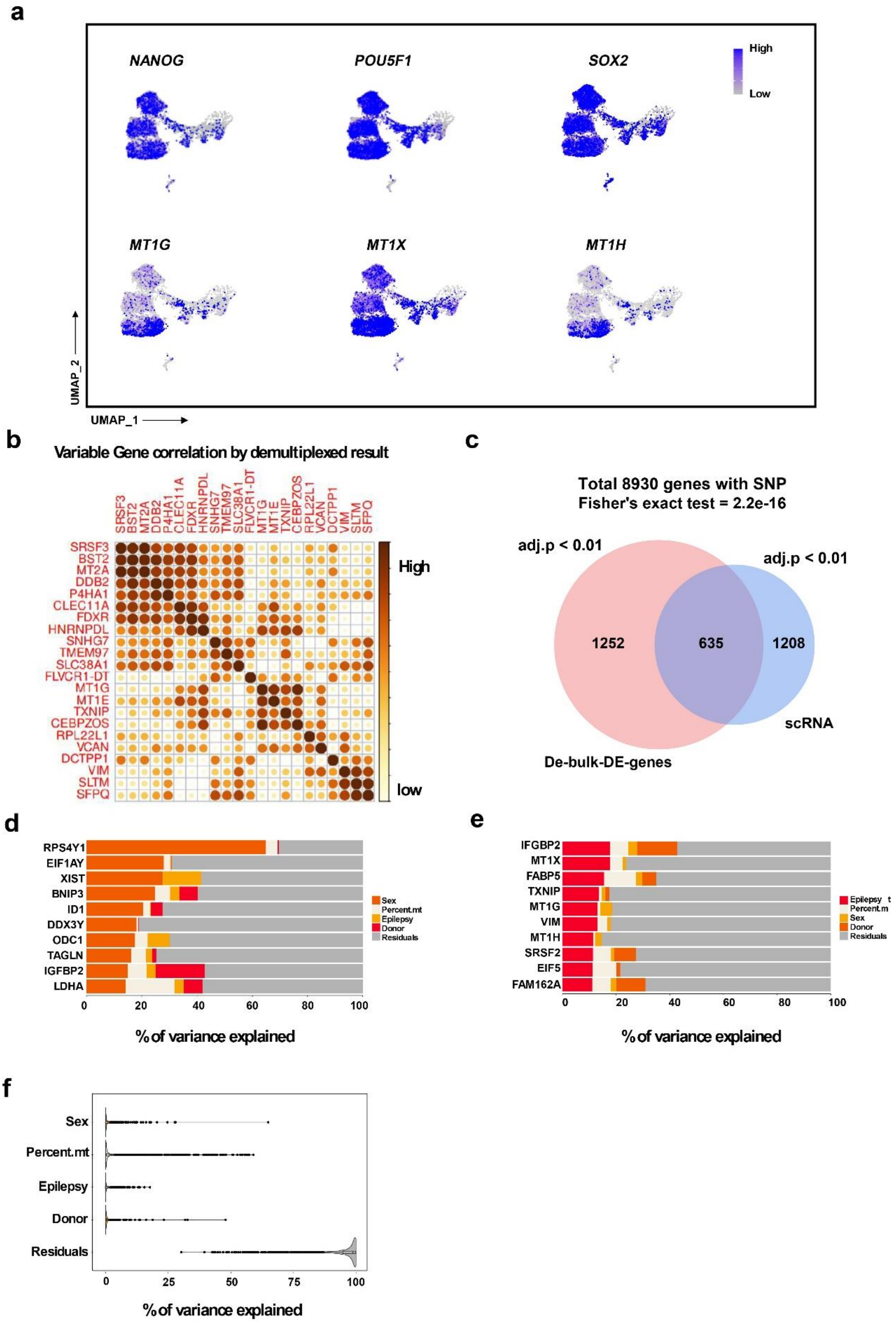

Supplementary Fig. 2 Supplmentary results of Vireo-bulk and variance analysis in iPSC scRNA-seq result. (a) Variable marker gene expression in single-cell RNA-seq results. (b) Gene correlation between Vireo-bulk (Y axis) and single-cell results

(X axis). (c) the adj.p is the cutoff of selected genes, and the P value of the Venn plot was calculated by the Fisher exact test. (d, e) Top 10 genes with the highest variance that are explained by sex or disease label across donors. (f) A violin plot for variance decomposition analysis in demultiplexed single-cell data. The X-axis indicates the percentage of variance that could be explained by variables listed on Y for each gene in variance analysis. Source data are provided as a Source Data file.

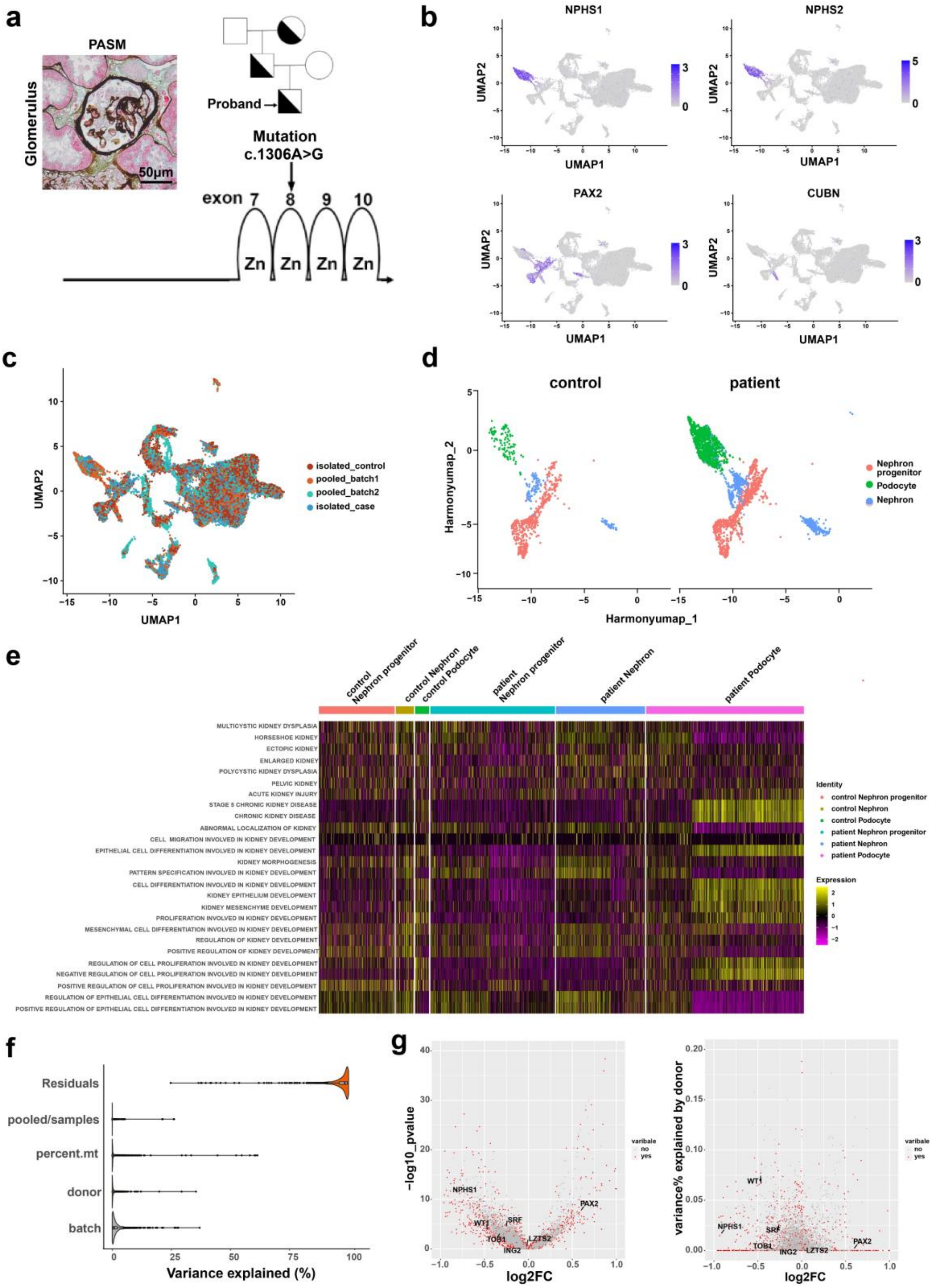

Supplementary Fig. 3. Supplymentary information of scRNA-seq results of kidney organoids samples. (a) Left panel: proband renal biopsy specimen. Right panel: Pedigrees of the proband and the *WT1* variant at c.1306A>G (p.R436G). (b)UMAP plots of marker genes of organoids. (c) UMAP plots for the 4 batches. (d) Subsets of cells in the chimeric organoid analysis, including distal nephrons, podocytes and nephrons. (e) Kidney-related pathway enrichment analysis of scRNA-seq results. Cells are labeled and clustered by donor source and cell type. (f) Violin plot for variance decomposition analysis in the single-cell demultiplexed results. The

x-axis indicates the percentage of variance that could be explained by variables listed on the y-axis for each gene in variance analysis. (g) Scatter plots of differential expression in scRNA-seq data (left panel; Variance Partion method) and variance decomposition analysis results (right panel; y-axis denotes variance explained by disease condition), where the x-axis represents the $\log_2$ fold change between disease and control. Source data are provided as a Source Data file.

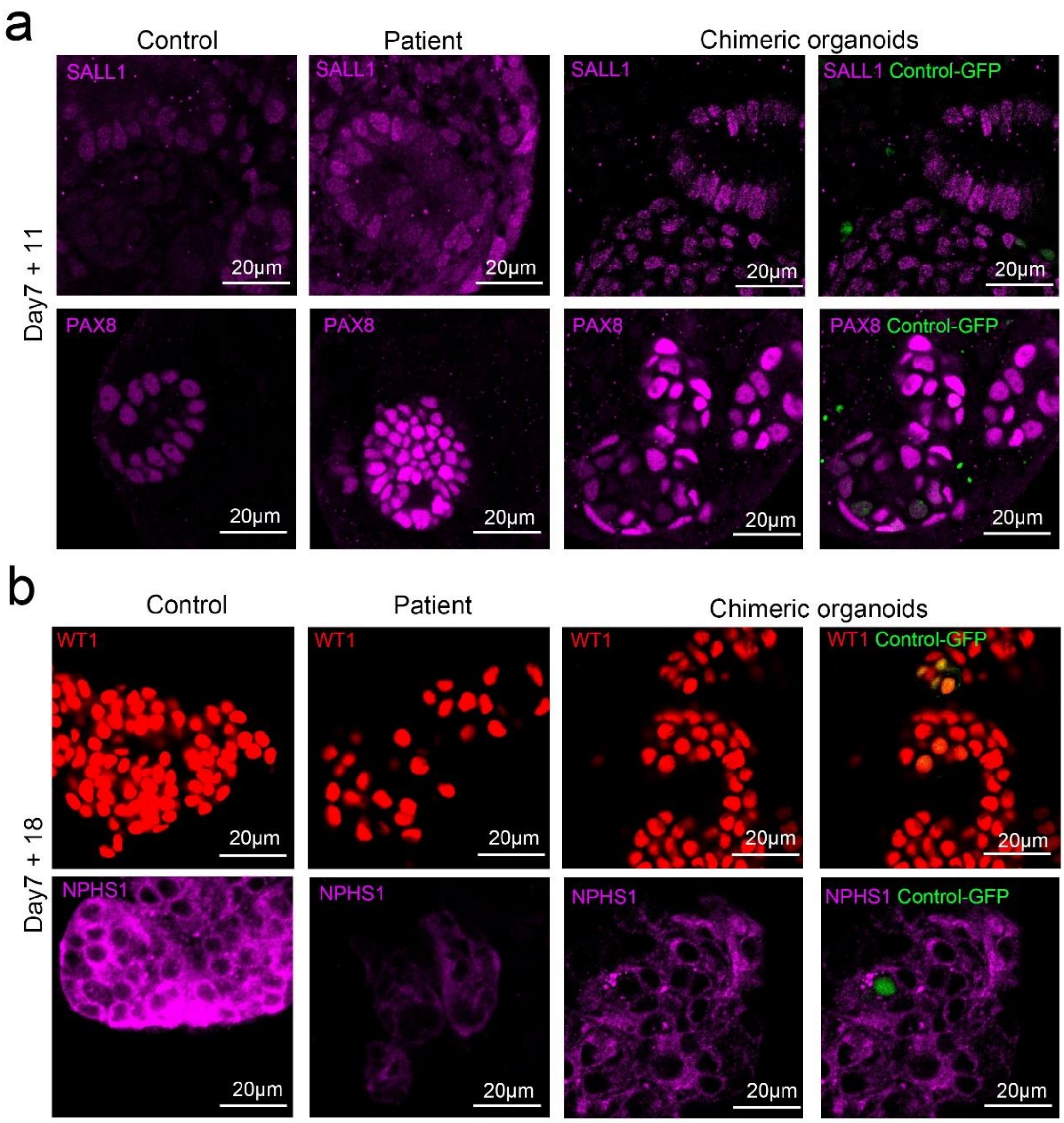

Supplementary Fig. 4. Chimeric kidney organoids demonstrated abnormal nephron development and podocyte-related proteins. (a) Confocal immunofluorescence images showing that nephron development markers SALL1, PAX8 protein expression level in the control, patient and chimeric kidney organoids. (b) Confocal immunofluorescence images showing that podocyte-related markers WT1, NPHS1 protein expression level in the control, patient and chimeric kidney organoids. Control-GFP: cells from differentiated control GFP-expressing iPSC cell line in the chimeric organoids. The experiment was repeated 3 times. Source data are provided as a Source Data file.
